# Manoalide Shows Mutual Interaction between Cellular and Mitochondrial Reactive Species with Apoptosis in Oral Cancer Cells

**DOI:** 10.1155/2021/6667355

**Published:** 2021-03-02

**Authors:** Hui-Ru Wang, Ping-Ho Chen, Jen-Yang Tang, Ching-Yu Yen, Yong-Chao Su, Ming-Yii Huang, Hsueh-Wei Chang

**Affiliations:** ^1^Department of Biomedical Science and Environmental Biology, PhD Program of Life Science, College of Life Science, Kaohsiung Medical University, Kaohsiung, Taiwan; ^2^School of Dentistry, College of Dental Medicine, Kaohsiung Medical University, Kaohsiung, Taiwan; ^3^Cancer Center, Kaohsiung Medical University Hospital, Kaohsiung, Taiwan; ^4^Department of Radiation Oncology, Kaohsiung Medical University Hospital, Kaohsiung, Taiwan; ^5^Department of Radiation Oncology, Faculty of Medicine, College of Medicine, Kaohsiung Medical University, Kaohsiung, Taiwan; ^6^Department of Oral and Maxillofacial Surgery, Chi-Mei Foundation Medical Center, Tainan, Taiwan; ^7^School of Dentistry, Taipei Medical University, Taipei, Taiwan; ^8^Center for Cancer Research, Kaohsiung Medical University, Kaohsiung, Taiwan

## Abstract

We previously found that marine sponge-derived manoalide induced antiproliferation and apoptosis of oral cancer cells as well as reactive species generations probed by dichloro-dihydrofluorescein diacetate (DCFH-DA) and MitoSOX Red. However, the sources of cellular and mitochondrial redox stresses and the mutual interacting effects between these redox stresses and apoptosis remain unclear. To address this issue, we examined a panel of reactive species and used the inhibitors of cellular reactive species (*N*-acetylcysteine (NAC)), mitochondrial reactive species (MitoTEMPO), and apoptosis (Z-VAD-FMK; ZVAD) to explore their interactions in manoalide-treated oral cancer Ca9-22 and CAL 27 cells. Hydroxyl (˙OH), nitrogen dioxide (NO_2_˙), nitric oxide (˙NO), carbonate radical-anion (CO_3_^˙–^), peroxynitrite (ONOO^–^), and superoxide (O_2_^˙–^) were increased in oral cancer cells following manoalide treatments in terms of fluorescence staining and flow cytometry. Cellular reactive species (˙OH, NO_2_^·^, ˙NO, CO_3_^˙–^, and ONOO^–^) as well as cellular and mitochondrial reactive species (O_2_^˙–^) were induced in oral cancer cells following manoalide treatment for 6 h. NAC, MitoTEMPO, and ZVAD inhibit manoalide-induced apoptosis in terms of annexin V and pancaspase activity assays. Moreover, NAC inhibits mitochondrial reactive species and MitoTEMPO inhibits cellular reactive species, suggesting that cellular and mitochondrial reactive species can crosstalk to regulate each other. ZVAD shows suppressing effects on the generation of both cellular and mitochondrial reactive species. In conclusion, manoalide induces reciprocally activation between cellular and mitochondrial reactive species and apoptosis in oral cancer cells.

## 1. Introduction

Oral cancer is a global disease with approximately 710,000 new cases of cancers of the oral cavity and pharynx per year and over 359,000 deaths worldwide [1]. In the world, cancers of the oral cavity and pharynx rank as the 7th more prevalent cancer and rank as the 9th cause of cancer death. To date, the incidence rate of oral and pharynx cancers among Taiwanese men ranks the highest worldwide [1, 2]. In Taiwan, cancers of the oral cavity and pharynx were the fourth most prevalent cancer among males [3]. At present, oral cancer is primarily treated by surgical resection, radiation therapy, chemotherapy, or a combination of the above therapies [4]. The anticancer drug development for oral cancer cells is still imperative.

Marine natural products are abundant resources for identifying anticancer drugs [5–8]. Manoalide, a marine sponge-derived sesterterpenoid [9], has antibiotic, analgesic, anti-inflammatory [10], and anticancer effects [11, 12]. In 1993, the first anticancer effect for antiproliferation by manoalide had been reported in human epidermoid carcinoma KB cells [13] but only the IC_50_ value was provided without investigating detailed mechanisms [11]. Manoalide has been used for Phase II (antipsoriatic) clinical trial but ceased by formulation problems [14].

Recently, we reported that manoalide exhibited antiproliferation, apoptosis, and DNA damage effects against oral cancer cells by inducing the cellular reactive species as probed by dichloro-dihydrofluorescein diacetate (DCFH-DA) [12]. However, the DCFH-DA was reported to be unreliable probe to detect H_2_O_2_ and other kind of ROS [15, 16]. Moreover, superoxide anion was reported to be incapable of crossing the mitochondrial membrane [17]. However, our previous study showed that MitoTEMPO (MT) [18], an mitochondrial superoxide (MitoSOX) inhibitor, suppressed manoalide-induced DNA damages (*γ*H2AX and 8-oxodG) [12], suggesting that MitoSOX may cross the mitochondrial membrane to induce DNA damage in oral cancer cells. Accordingly, the MitoSOX traffic to exit mitochondria is controversial. Therefore, the traffic between manoalide-induced cellular and mitochondrial reactive species remains unclear. It warrants for detailed investigation for the involvement of more different cellular and mitochondrial reactive species after manoalide treatment.

In the present study, we aimed to determine the changes of several types of reactive species using several available probes [19] in oral cancer Ca9-22 and CAL 27 cells following manoalide treatment. Levels of the cellular reactive species such as nitrogen dioxide (NO_2_^˙^), carbonate radical-anion (CO_3_^˙–^), hydroxyl (˙OH), peroxynitrite (ONOO^–^), and nitric oxide (˙NO) as well as the cellular and mitochondrial superoxide (O_2_^˙–^) were estimated.

Using the inhibitors for cellular and mitochondrial oxidative stresses (*N*-acetylcysteine (NAC) and MitoTEMPO (MT)), the sources of cellular and mitochondrial reactive species and its apoptosis-modulating effect in oral cancer cells after manoalide treatment were analyzed. Using the inhibitors for apoptosis (Z-VAD-FMK; ZVAD), the cellular and mitochondrial reactive species-modulating effect of apoptosis in oral cancer cells after manoalide treatment was explored. Therefore, the possibility that manoalide induced the mutual interaction between cellular and mitochondrial reactive species and apoptosis in oral cancer cells were examined in the current study.

## 2. Materials and Methods

### 2.1. Cell Culture, Cell Viability, Apoptosis, Manoalide, and Inhibitors

The human oral cancer cell lines (Ca9-22 and CAL 27), collected from Health Science Research Resources Bank (HSRRB; Osaka, Japan) and American Type Culture Collection (ATCC; Manassas, VA, USA), were maintained in DMEM formula (Gibco, Grand Island, NY, USA) with 10% fetal bovine serum as previously described [20]. Cell viability for 6 h manoalide treatment (10 *μ*M) was determined by MTS assay [12]. Apoptosis was determined by both annexin V/7-aminoactinmycin D (7AAD) (Strong Biotech Corporation, Taipei, Taiwan) and pancaspase activity (Abcam, Cambridge, UK) [21] assays as previously described.

Manoalide, mitochondrial superoxide inhibitor MT [18] (Cayman Chemical, Ann Arbor, MI, USA), and panapoptosis inhibitor ZVAD [22] (http://Selleckchem.com/; Houston, TX, USA) were dissolved in DMSO. A cellular reactive species inhibitor NAC [23, 24] (Sigma-Aldrich; St Louis, MO, USA) was dissolved in double distilled water.

### 2.2. Probes for Several Reactive Species

Measurements for several reactive species could be detected using several probes (Sigma, St Louis, MO, USA) as follows [19]. DCFH-DA is a probe for NO_2_˙, CO_3_˙¯, and ˙OH. Hydroxyphenyl fluorescein (HPF) is a probe for ˙OH and ONOO-. 4-amino-5-methylamino-2′,7′-difluorofluorescein (DAF-FM) is a probe for ˙NO. Dihydroethidium (DHE) and MitoSOX Red are probes for cellular and mitochondria O_2_˙¯, respectively [16]. These probes were dissolved in DMSO and all experiments with or without probes had the same concentration of 0.1% DMSO.

### 2.3. Fluorescence Staining for Several Reactive Species

After manoalide treatment for 6 h, cells were stained with DCFH-DA (10 mM, 30 min), DHE (50 *μ*M, 30 min), MitoSOX Red (2.5 mM, 10 min), DAF-FM (100 *μ*M, 30 min), or HPF (100 *μ*M, 30 min) [19] and washed with 1x PBS before microscopy. DCFH-DA, DAF-FM, and HPF were observed by Leica DMi8 fluorescence microscope at excitation (ex)/emission (em) 488/525 nm while DHE and MitoSOX Red were observed by Olympus FV1000 confocal microscope at ex/em for 405/605 nm [25].

### 2.4. Flow Cytometry for Several Reactive Species

After manoalide treatment for 6 h, cells were stained with DCFH-DA [26] (1 mM, 30 min), DHE [27] (5 *μ*M, 30 min), MitoSOX Red [28] (0.25 mM, 10 min), DAF-FM [29] (10 *μ*M, 30 min), or HPF [30] (10 *μ*M, 30 min), and washed with 1x PBS. Subsequently, DCFH-DA, DAF-FM, and HPF were observed by Guava® easyCyte flow cytometer (Merck KGaA; Darmstadt, Germany) at ex/em for 488/525 nm. DHE and MitoSOX Red were observed by LSR II flow cytometer (Becton-Dickinson, Mansfield, MA, USA) at ex/em for 405/585 nm to avoid the nonspecific superoxide detection under 488 nm excitation [25]. Data were analyzed by Flow Jo (FlowJo LLC, Ashland, OR, USA).

To evaluate the suppression powder of inhibitors on manoalide-induced reactive species, we use the formula of suppression (fold) to calculate as follows: Suppression fold of reactive species inhibitors = (mean intensity of manoalide / mean intensity of control) / (mean intensity of inhibitors and manoalide / mean intensity of inhibitors), where inhibitors can be NAC, MT, and ZVAD. When manoalide concentration is zero, the suppression (fold) of inhibitor is 1.

### 2.5. Statistics

The significance of the difference in multiple comparisons were analyzed by one-way ANOVA with Tukey HSD post hoc test (JMP® 12 software). Results from different treatments are considered significantly different for multiple comparison (indicated via different letters without overlapping) if *p* < 0.05.

## 3. Results

### 3.1. Several Reactive Species Were Detectable in Oral Cancer Cells after Manoalide Treatment

**Figure 1:**
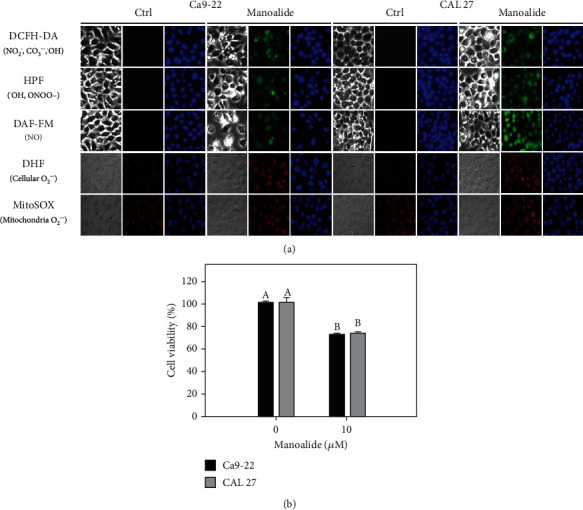
Detection of several kinds of reactive species and cell viability in oral cancer cells after 6 h manoalide treatment. Cells were treated under control (0.1% DMSO only) and manoalide (10 *μ*M) treatments for 6 h. All experiments had the same concentration of DMSO. (a) Fluorescence staining images of cellular and mitochondrial radical probes with DCFH-DA, HPF, DAF-FM, DHE, and MitoSOX Red in manoalide-treated oral cancer (Ca9-22 and CAL 27) cells. For each treatment, the light microscope, radical probe, and Hoechst 33342 (2′-[4-ethoxyphenyl]-5-[4-methyl-1-piperazinyl]-2,5′-bi-1H-benzimidazole trihydrochloride trihydrate) counterstaining images were provided. DCFH-DA-, HPF-, and DAF-FM-probed images were captured by Leica DMi8 fluorescence microscope. DHE- and MitoSOX-probed images were captured by Olympus FV1000 confocal microscope. (b) MTS assay for cell viability determination. Results between control and manoalide treatment of the same cells are considered significantly different (indicated via different letters without overlapping) (*p* < 0.0001). Data, means ± SDs (*n* = 3 independent experiments, each experiment was performed with three replications).

As shown in Figure 1(a), cellular reactive species (NO_2_^˙^, CO_3_^˙–^, ˙OH, ONOO^–^, ˙NO, and O_2_^˙–^) in manoalide-treated oral cancer cells were detected by using available probes such as DCFH-DA, HPF, DAF-FM, and DHE). Mitochondrial O_2_^˙–^ reactive species was probed by MitoSOX Red. Both cellular and mitochondrial reactive species showed positive fluorescent staining in oral cancer Ca9-22 and CAL 27 cells after manoalide treatment (10 *μ*M, 6 h), demonstrating that the radical probes DCFH-DA, HPF, DAF-FM, DHE, and MitoSOX Red were able to qualitatively detect their reactive species. The relative quantitative analyses were analyzed by flow cytometer as shown in the following experiments. Under manoalide treatment (10 *μ*M, 6 h), oral cancer Ca9-22 and CAL 27 cells showed about 76% cell viability (Figure 1(b)), suggesting short-term exposure of manoalide exhibited a detectable cell killing effect to oral cancer cells.

### 3.2. Several Reactive Species Were Differentially Generated in Oral Cancer Cells after Manoalide Treatment

**Figure 2:**
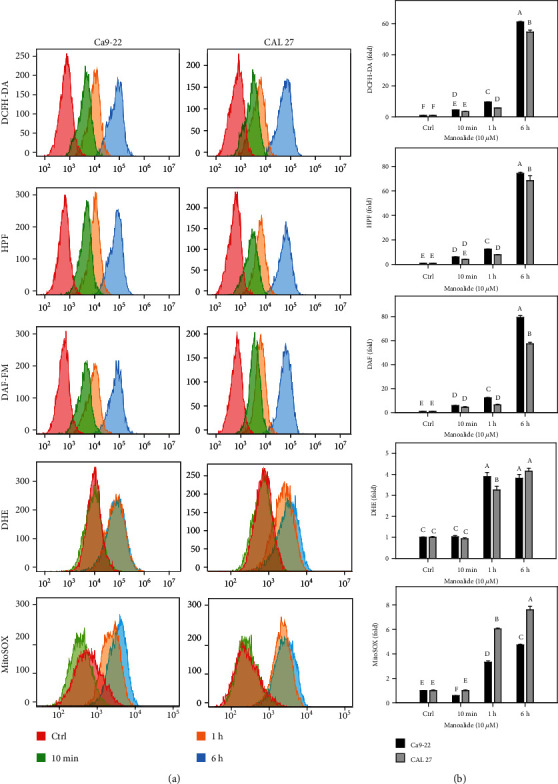
Flow cytometry of radical probes with DCFH-DA, HPF, DAF-FM, DHE, and MitoSOX Red in manoalide-treated oral cancer cells. Cells were treated with control (0.1% DMSO only) and manoalide (10 *μ*M) for 0, 10 min, 1 h, and 6 h. All experiments had the same concentration of DMSO. (a) Flow cytometry patterns for manoalide-treated oral cancer cells (Ca9-22 and CAL 27). (b) Statistics. The reactive mean intensity for the control is set to 1. Results from different treatments are considered significantly different for multiple comparison (indicated via different letters without overlapping) (*p* < 0.05 to 0.0001). In the example of (b), the DCFH-DA fold for control, 10 min, 1 h, and 6 h show letters at top for “f,” “de,” “c,” and “a” for Ca9-22 cells. Since they were marked with different letters without overlapping, all the treatments between each other for control, 10 min, 1 h, and 6 h differ significantly. Moreover, the DCFH-DA fold at 6 h for Ca9-22 and CAL 27 cells show letters at top for “a” and “b,” indicating that they are significantly different. Data, means ± SDs (*n* = 3 independent experiments, each experiment collected with 10000 gated cell counts).

Using flow cytometry, the levels of several reactive species were measured in manoalide-treated oral cancer cells by using available probes (Figure 2(a)). Since free radicals were short-lived intermediates [16, 31], all test probes (DCFH-DA, HPF, DAF-FM, DHE, and MitoSOX Red) were detected in short time (0, 10 min, 1 h, and 6 h). These test probes showed differential increase for their corresponding reactive radicals in a time-dependent manner to oral cancer Ca9-22 and CAL 27 cells after manoalide treatment (10 *μ*M) (Figure 2(b)). Moreover, cellular reactive species (probed by DCFH-DA, HPF, DAF-FM, and DHE) and mitochondrial reactive species (probed by MitoSOX Red) were differentially induced in manoalide-treated oral cancer cells. Since 6 h manoalide treatment (10 *μ*M) showed the highest intensity for all reactive species ranging from 0 to 6 h, the following experiments were performed according to this condition.

### 3.3. Manoalide-Induced Apoptosis Was Differentially Suppressed by NAC, MT, and ZVAD in Oral Cancer Cells

**Figure 3:**
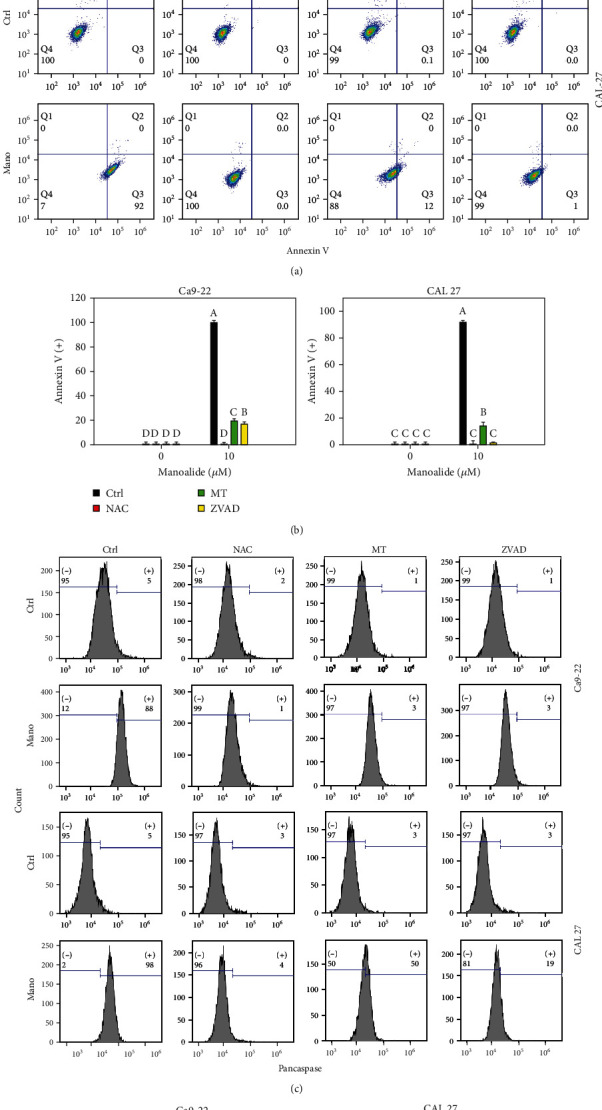
Apoptosis effects of inhibitors for cellular reactive species, mitochondrial reactive species, and apoptosis (NAC, MT, and ZVAD) in manoalide-treated oral cancer cells. Cells (Ca9-22 and CAL 27) were pretreated with control (0.1% DMSO only), NAC (8 mM), MT (20 *μ*M), and ZVAD (100 *μ*M) for 2 h and posttreated with control and manoalide (10 *μ*M) for 0 and 6 h. All experiments had the same concentration of DMSO. (a) Flow cytometry patterns of annexin V/7AAD staining for manoalide-treated oral cancer cells. (b) Statistics of (a). (c) Flow cytometry patterns of pancaspase activity for manoalide-treated oral cancer cells. (d) Statistics of (c). Results from different inhibitor treatments are considered significantly different compared to control (indicated via different letters without overlapping) (*p* < 0.05 to 0.0001). Data, means ± SDs (*n* = 3 independent experiments, each experiment collected with 10000 gated cell counts).

Following pretreatments of inhibitors for cellular reactive species, mitochondrial reactive species, and apoptosis, i.e., *N*-acetylcysteine (NAC), MitoTEMPO (MT), and apoptosis (ZVAD), the flow cytometry patterns of annexin V/7AAD and pancaspase activity-detected apoptosis in manoalide (10 *μ*M, 6 h) posttreated oral cancer Ca9-22 and CAL 27 cells were provided (Figures 3(a) and 3(c)). As shown in Figures 3(b) and 3(d), annexin V- and pancaspse activity-detected apoptosis was highly induced by manoalide in oral cancer cells, which was suppressed by pretreatments of NAC, MT, and ZVAD.

### 3.4. DCFH-DA-Detected Cellular Reactive Species Were Differentially Suppressed by NAC, MT, and ZVAD in Manoalide-Treated Oral Cancer Cells

**Figure 4:**
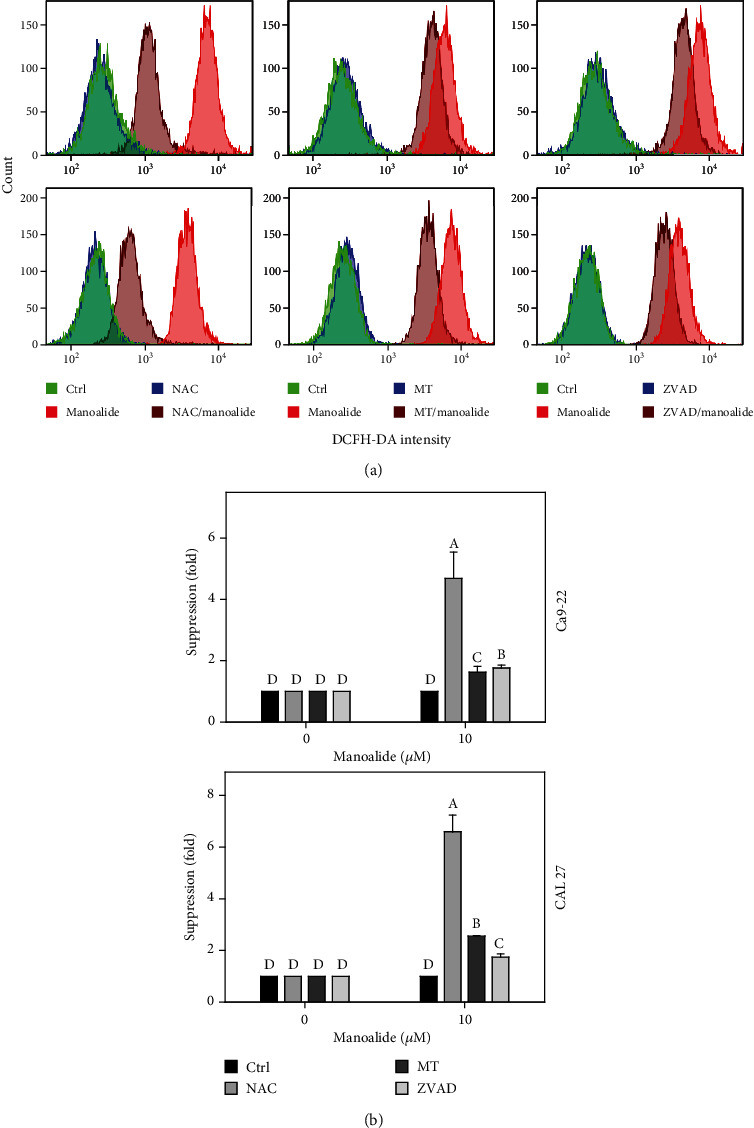
Effects of inhibitors for cellular reactive species, mitochondrial reactive species, and apoptosis (NAC, MT, and ZVAD) on flow cytometry of DCFH-DA (NO_2_˙, CO_3_˙¯, and ˙OH) in manoalide-treated oral cancer cells. Cells were pretreated with control (0.1% DMSO only), NAC (8 mM), MT (20 *μ*M), and ZVAD (100 *μ*M) for 2 h and posttreated with control and manoalide (10 *μ*M) for 0 and 6 h. All experiments had the same concentration of DMSO. (a) Flow cytometry patterns for manoalide-treated oral cancer cells (Ca9-22 and CAL 27). (b) Statistics of suppression (fold). The suppression fold is defined in detail at Section 2.4. No suppression is defined at 1 (untreated control; 0.1% DMSO only). If the reactive species intensity determined by flow cytometry is decreased after inhibitor treatment, the suppression fold of inhibitors (NAC, MT, and ZVAD) is larger than 1. Results from different treatments are considered significantly different for multiple comparisons (indicated via different letters without overlapping) (*p* < 0.0001). In the example of Ca9-22 cells, the suppression fold for control, NAC, MT, and ZVAD show letters at top for “D,” “A,” “C,” and “B” for Ca9-22 cells. Since they were marked with different letters without overlapping, all the treatments between each other (control, NAC, MT, or ZVAD) differ significantly. Data, means ± SDs (*n* = 3 independent experiments, each experiment collected with 10000 gated cell counts).

Following pretreatments of inhibitors for cellular reactive species, mitochondrial reactive species, and apoptosis, i.e., NAC, MT, and ZVAD, the flow cytometry patterns of DCFH-DA-detected reactive species in manoalide posttreated oral cancer Ca9-22 and CAL 27 cells were provided (Figure 4(a)). As shown in Figure 4(b), DCFH-DA-detected cellular reactive species were highly induced by manoalide in oral cancer cells, which were suppressed by pretreatments of NAC, MT, and ZVAD.

### 3.5. HPF-Detected Cellular Reactive Species Were Differentially Suppressed by NAC, MT, and ZVAD in Manoalide-Treated Oral Cancer Cells

**Figure 5:**
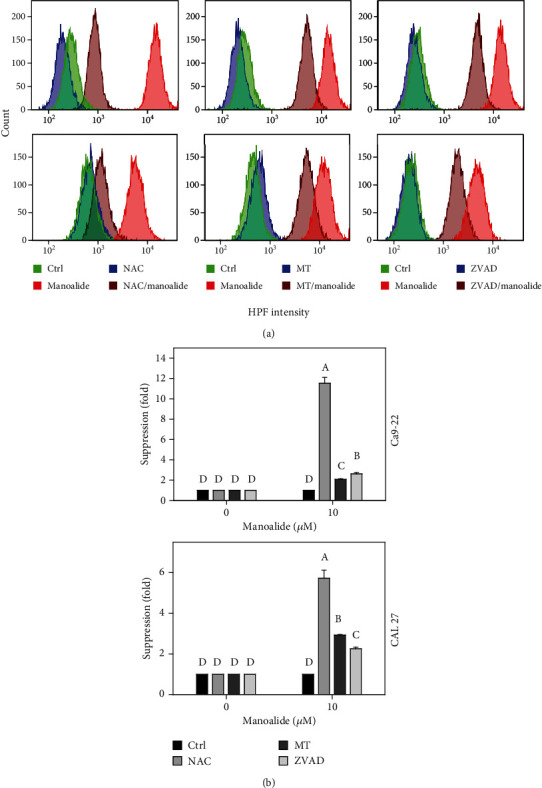
Effects of inhibitors for cellular reactive species, mitochondrial reactive species, and apoptosis (NAC, MT, and ZVAD) on flow cytometry of HPF (˙OH and ONOO-) in manoalide-treated oral cancer cells. Cells were pretreated with control (0.1% DMSO only), NAC (8 mM), MT (20 *μ*M), and ZVAD (100 *μ*M) for 2 h and posttreated with control and manoalide (10 *μ*M) for 0 and 6 h. All experiments had the same concentration of DMSO. (a) Flow cytometry patterns for manoalide-treated oral cancer cells (Ca9-22 and CAL 27). (b) Statistics of suppression (fold). The suppression fold is defined in detail at Section 2.4. No suppression is defined at 1 (untreated control; 0.1% DMSO only). If the reactive species intensity determined by flow cytometry is decreased after inhibitor treatment, the suppression fold of inhibitors (NAC, MT, and ZVAD) is larger than 1. Results from different treatments are considered significantly different for multiple comparison (indicated via different letters without overlapping) (*p* < 0.0001). Data, means ± SDs (*n* = 3 independent experiments, each experiment collected with 10000 gated cell counts).

Following pretreatments of NAC, MT, and ZVAD, the flow cytometry patterns of HPF-detected reactive species in manoalide posttreated oral cancer Ca9-22 and CAL 27 cells were provided (Figure 5(a)). As shown in Figure 5(b), HPF-detected cellular reactive species were highly induced by manoalide in oral cancer cells, which were suppressed by pretreatments of NAC, MT, and ZVAD.

### 3.6. DAF-FM-Detected Cellular Reactive Species Were Differentially Suppressed by NAC, MT, and ZVAD in Manoalide-Treated Oral Cancer Cells

**Figure 6:**
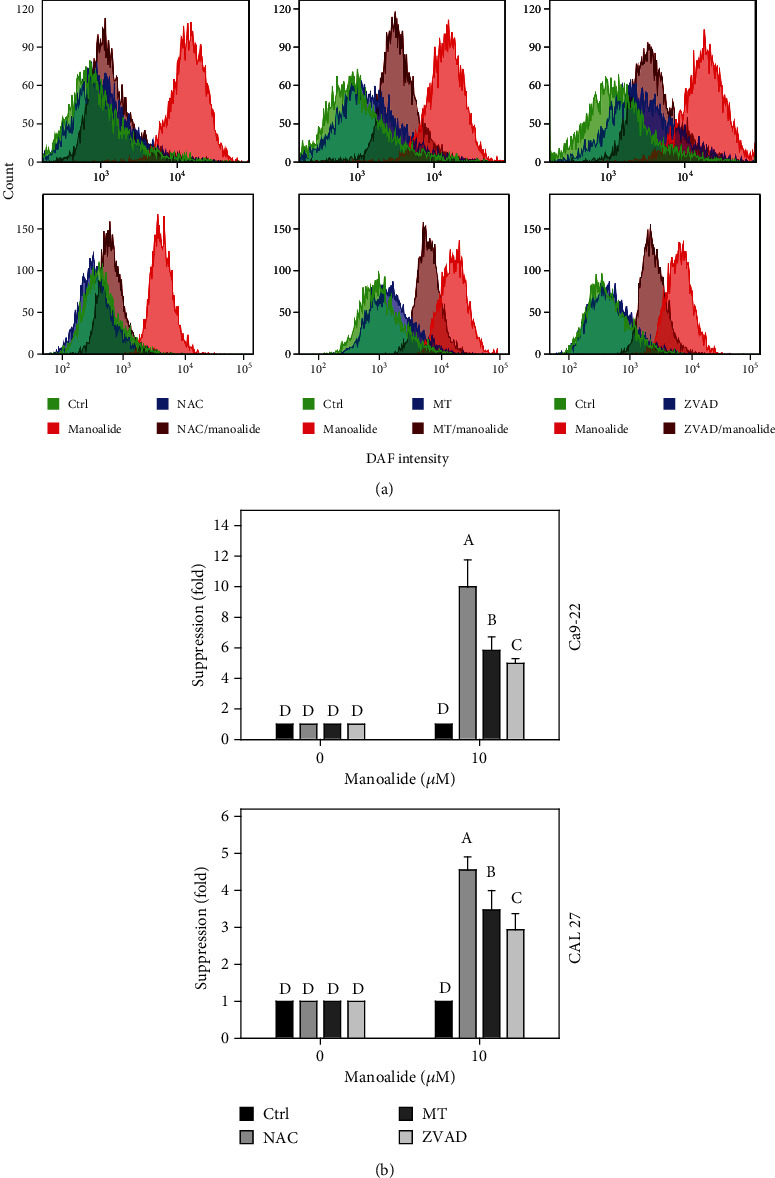
Effects of inhibitors for cellular reactive species, mitochondrial reactive species, and apoptosis (NAC, MT, and ZVAD) on flow cytometry of DAF-FM (˙NO) in manoalide-treated oral cancer cells. Cells were pretreated with control (0.1% DMSO only), NAC (8 mM), MT (20 *μ*M), and ZVAD (100 *μ*M) for 2 h and posttreated with control and manoalide (10 *μ*M) for 0 and 6 h. All experiments had the same concentration of DMSO. (a) Flow cytometry patterns for manoalide-treated oral cancer cells (Ca9-22 and CAL 27). (b) Statistics of suppression (fold). The suppression fold is defined in detail at Section 2.4. No suppression is defined at 1 (untreated control; 0.1% DMSO only). If the reactive species intensity determined by flow cytometry is decreased after inhibitor treatment, the suppression fold of inhibitors (NAC, MT, and ZVAD) is larger than 1. Results from different treatments are considered significantly different for multiple comparison (indicated via different letters without overlapping) (*p* < 0.01 to 0.0001). Data, means ± SDs (*n* = 3 independent experiments, each experiment collected with 10000 gated cell counts).

Following pretreatments of NAC, MT, and ZVAD, the flow cytometry patterns of DAF-FM-detected reactive species in manoalide posttreated oral cancer Ca9-22 and CAL 27 cells were provided (Figure 6(a)). As shown in Figure 6(b), DAF-FM-detected cellular reactive species were highly induced by manoalide in oral cancer cells, which were suppressed by pretreatments of NAC, MT, and ZVAD.

### 3.7. DHE-Detected Cellular Reactive Species Were Differentially Suppressed by NAC, MT, and ZVAD in Manoalide-Treated Oral Cancer Cells

**Figure 7:**
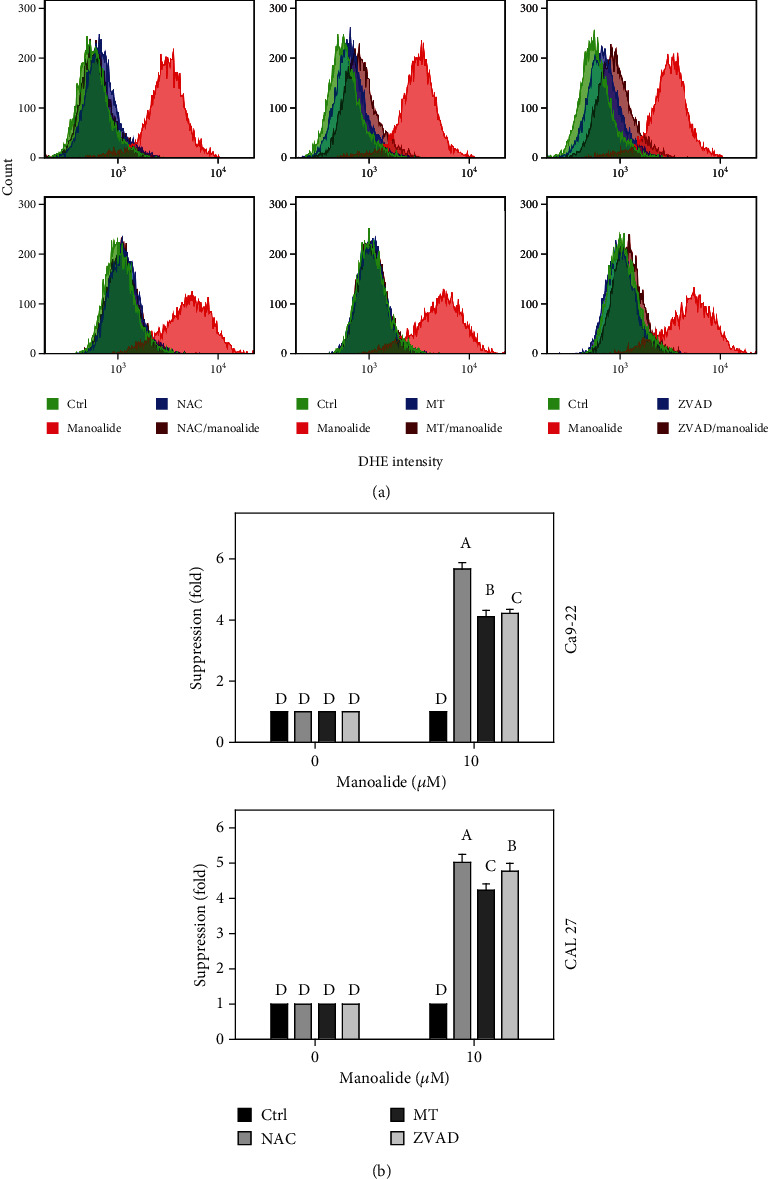
Effects of inhibitors for cellular reactive species, mitochondrial reactive species, and apoptosis (NAC, MT, and ZVAD) on flow cytometry of DHE (cellular O_2_˙¯) in manoalide-treated oral cancer cells. Cells were pretreated with control (0.1% DMSO only), NAC (8 mM), MT (20 *μ*M), and ZVAD (100 *μ*M) for 2 h and posttreated with control and manoalide (10 *μ*M) for 0 and 6 h. All experiments had the same concentration of DMSO. (a) Flow cytometry patterns for manoalide-treated oral cancer cells (Ca9-22 and CAL 27). (b) Statistics of suppression (fold). The suppression fold is defined in detail at Section 2.4. No suppression is defined at 1 (untreated control; 0.1% DMSO only). If the reactive species intensity determined by flow cytometry is decreased after inhibitor treatment, the suppression fold of inhibitors (NAC, MT, and ZVAD) is larger than 1. Results from different treatments are considered significantly different for multiple comparison (indicated via different letters without overlapping) (*p* < 0.05 to 0.0001). Data, means ± SDs (*n* = 3 independent experiments, each experiment collected with 10000 gated cell counts).

Following pretreatments of NAC, MT, and ZVAD, the flow cytometry patterns of DHE-detected reactive species in manoalide posttreated oral cancer Ca9-22 and CAL 27 cells were provided (Figure 7(a)). As shown in Figure 7(b), DHE-detected cellular O_2_^˙–^ reactive species were highly induced by manoalide in oral cancer cells, which were suppressed by pretreatments of NAC, MT, and ZVAD.

### 3.8. MitoSOX Red-Detected Mitochondrial Reactive Species Were Differentially Suppressed by NAC, MT, and ZVAD in Manoalide-Treated Oral Cancer Cells

**Figure 8:**
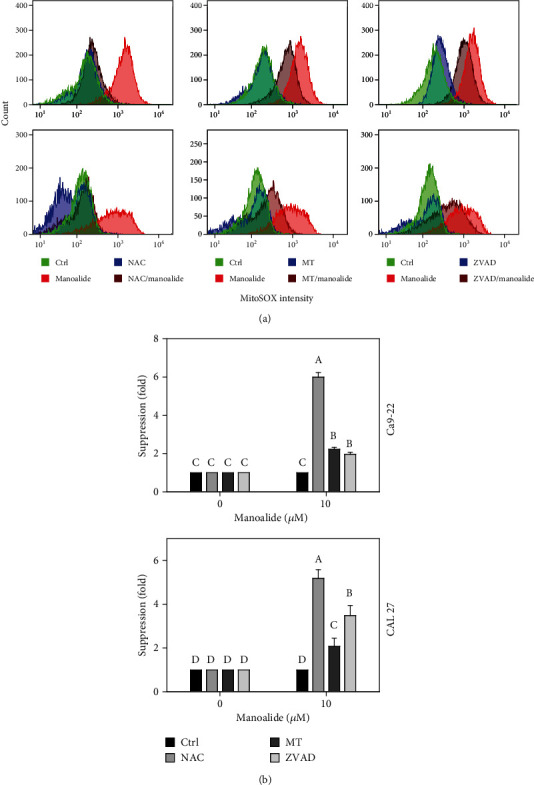
Effects of inhibitors for cellular reactive species, mitochondrial reactive species, and apoptosis (NAC, MT, and ZVAD) on flow cytometry of MitoSOX Red (mitochondrial O_2_˙¯) in manoalide-treated oral cancer cells. Cells were pretreated with control (0.1% DMSO only), NAC (8 mM), MT (20 *μ*M), and ZVAD (100 *μ*M) for 2 h and posttreated with control and manoalide (10 *μ*M) for 0 and 6 h. All experiments had the same concentration of DMSO. (a) Flow cytometry patterns for manoalide-treated oral cancer cells (Ca9-22 and CAL 27). (b) Statistics of suppression (fold). The suppression fold is defined in detail at Section 2.4. No suppression is defined at 1 (untreated control; 0.1% DMSO only). If the reactive species intensity determined by flow cytometry is decreased after inhibitor treatment, the suppression fold of inhibitors (NAC, MT, and ZVAD) is larger than 1. Results from different treatments are considered significantly different for multiple comparison (indicated via different letters without overlapping) (*p* < 0.0001). Data, means ± SDs (*n* = 3 independent experiments, each experiment collected with 10000 gated cell counts).

Following pretreatments of NAC, MT, and ZVAD, the flow cytometry patterns of MitoSOX Red-detected reactive species in manoalide posttreated oral cancer Ca9-22 and CAL 27 cells were provided (Figure 8(a)). As shown in Figure 8(b), MitoSOX Red-detected mitochondrial O_2_^˙–^ reactive species were highly induced by manoalide in oral cancer cells, which were suppressed by pretreatments of NAC, MT, and ZVAD.

## 4. Discussion

Drugs with redox-modulating ability have the potential for selective killing on cancer cells [32–34]. Manoalide was validated to have this redox-modulating ability for selective killing on oral cancer cells [12]; however, its redox evidence of manoalide relies on DCFH-DA and MitoSOX Red-detected reactive species. Moreover, the DCFH-DA was reported to be unreliable probe to detect H_2_O_2_ (˙OH) [15, 16]. DCFH-DA also crossdetected NO_2_˙ and CO_3_˙¯. Accordingly, more probes detecting other reactive species as indicated in Figure 1 are necessary to clarify the redox-modulating ability of manoalide.

In the present study, we investigated the sources of cellular and mitochondrial oxidative stresses in oral cancer cells after manoalide treatment. Moreover, the interaction among these manoalide-induced reactive species and apoptosis in oral cancer cells were explored.

### 4.1. Cellular Reactive Species May Regulate Mitochondrial Reactive Species

Based on the finding using the inhibitor pretreatment (NAC) of cellular reactive species, the manoalide-induced cellular reactive species as probed by DCFH-DA, HPF, DAF-FM, and DHE were suppressed (Figures 4–7). Similarly, FasL-stimulated cellular reactive species as probed by dihydrorhodamine (DHR for H_2_O_2_ detection), HPF, and DHE were suppressed by NAC in Jurkat cells [35]. Thrombin-induced cellular reactive species as probed by DHE was suppressed by NAC in platelets *in vitro* [27]. Moreover, NAC pretreatment also suppressed the mitochondrial reactive species (MitoSOX) (Figure 8). Similarly, antimycin A [36] and withanolide C [37] -induced MitoSOX generations were suppressed by NAC in oral and breast cancer cells, respectively. Therefore, cellular reactive species may induce mitochondrial reactive species generations in oral cancer cells.

### 4.2. Mitochondrial Reactive Species May Regulate Cellular Reactive Species

Superoxide may be derived from the sources of NAPDH oxidase (NOX) [38] and mitochondria [39]. For mitochondria, complexes I and III are responsible for continuously producing reactive species during electron transfer [40]. Mitochondrial superoxide was reported to be highly membrane impermeable [41], which was supported by the finding that complex I-dependent superoxide is exclusively fluxed to matrix without escaping from mitochondria to cytoplasm [42]. However, this team also reported that that complex III can release superoxide to both the matrix and outer mitochondrial membrane [42], which may partly release to cytoplasm. Therefore, the traffic of mitochondrial reactive species to exit mitochondria is controversial.

Based on our findings using the inhibitor pretreatment (MT) of mitochondrial reactive species, the manoalide-induced mitochondrial reactive species as probed by MitoSOX Red were suppressed (Figure 8). Moreover, MT pretreatment also suppressed cellular reactive species as probed by DCFH-DA, HPF, DAF-FM, and DHE (Figures 4–7). Therefore, manoalide-induced mitochondrial reactive species may induce cellular reactive species generations in oral cancer cells, suggesting that mitochondrial reactive species may exit from mitochondria to cytoplasm to regulate the cellular reactive species.

### 4.3. Both Cellular and Mitochondrial Reactive Species May Regulate Apoptosis

At 6 h manoalide treatment, apoptosis is triggered in oral cancer Ca9-22 and CAL 27 cells. This manoalide-induced apoptosis was differentially suppressed by NAC, MT, and ZVAD in oral cancer cells (Figure 3). In addition to radical species scavenging (Figures 4–8), both NAC and MT can inhibit apoptosis after manoalide treatment in oral cancer cells. Therefore, cellular and mitochondrial reactive species can induce manoalide-induced apoptosis.

### 4.4. Apoptosis May Regulate Both Cellular and Mitochondrial Reactive Species

It is well known that reactive species can induce apoptosis. However, the role of apoptosis in the induction of reactive species is rarely investigated. ZVAD, a common pancaspase inhibitor to suppress apoptosis, was used to investigate the modulating effect of apoptosis to reactive species response. For example, ZVAD inhibits etoposide-induced caspase activation and DCFH-DA-detected reactive species generation in cervical cancer HeLa cells [43]. ZVAD inhibits cytosine analogue ferropoptoside N69-induced DCFH-DA-detected reactive species generation in melanoma cells [44]. ZVAD also suppresses oxidized black carbon-induced DCFH-DA detected reactive species generation in lung cancer cells [45]. These studies suggest that several drug-induced apoptosis can induce DCFH-DA-detected reactive species.

Similarly, we found that manoalide induced a number of cellular (Figures 4–7) and mitochondrial (Figure 8) reactive species generations, which were suppressed by ZVAD pretreatment in oral cancer cells. It shows that manoalide induces a caspase-dependent reactive species generation in oral cancer cells. These results suggest that apoptosis may trigger both manoalide-induced cellular and mitochondrial reactive species generations. Therefore, manoalide can induce apoptosis as well as cellular and mitochondrial reactive species in oral cancer cells, and they have the reciprocal activation between each other.

Moreover, many drug-induced apoptosis in cancer cell studies [46–49] also triggered oxidative stress but these studies only relied on the cellular reactive species detection by DCFH-DA. Our finding demonstrates that more cellular and mitochondrial reactive species as probed by DCFH-DA HPF, DAF-FM, DHE, and MitoSOX red also contribute to manoalide-induced redox changes to induce apoptosis. Therefore, multiple kinds of cellular and mitochondrial reactive species are suggested to be considered in drug-induced apoptosis studies.

## 5. Conclusions

**Figure 9:**
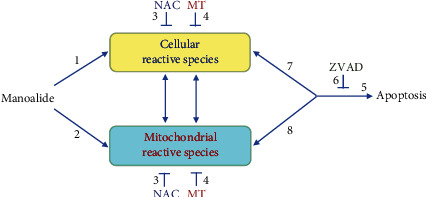
Expected mechanism of multifaceted inductions for cellular and mitochondrial reactive species to apoptosis on manoalide-treated oral cancer cells. NAC is an inhibitor for cellular reactive species (as probed by DCFH-DA, HPF, DAF-FM, and DHE), MT is an inhibitor for mitochondrial reactive species (as probed by MitoSOX Red), and ZVAD is an inhibitor for apoptosis. We proposed a possible mechanism that manoalide (10 *μ*M, 6 h) can induce (1) cellular and (2) mitochondrial reactive species. Moreover, (3) NAC inhibits manoalide-induced mitochondrial reactive species and (4) MT inhibits manoalide-induced cellular reactive species, suggesting that cellular and mitochondrial reactive species can reciprocally induce each other in manoalide-treated oral cancer cells. (5) Manoalide induces apoptosis, which are suppressed by (3) NAC, (4) MT, and (6) ZVAD, suggesting that (7) cellular and (8) mitochondrial radical species can trigger apoptosis. Interestingly, (6) ZVAD also inhibits both (7) cellular and (8) mitochondrial reactive species, suggesting that apoptosis may induce manoalide-induced cellular and mitochondrial reactive species in oral cancer cells. Therefore, manoalide exhibits reciprocally activation between cellular reactive species, mitochondrial reactive species, and apoptosis in oral cancer cells. Note: arrow and T symbol indicate the activating and inhibiting effects. DCFH-DA is the probe for NO_2_˙, CO_3_˙¯, and ˙OH. HPF is the probe for ˙OH and ONOO-. DAF-FM is the probe for ˙NO. DHE and MitoSOX Red are the probes for cellular and mitochondrial O_2_˙¯.

In the present study, manoalide (10 *μ*M, 6 h) induces DCFH-DA, HPF, DAF-FM, and DHE detected cellular reactive species and induces MitoSOX Red-detected mitochondrial reactive species, which are respectively inhibited by NAC and MT pretreatment. These cellular reactive species induce MitoSOX Red-detected mitochondrial reactive species, which are validated by the presence of NAC. These mitochondrial reactive species may induce cellular reactive species, which is validated by the presence of MT. It shows the reciprocally activation between cellular and mitochondrial reactive species after manoalide treatment in oral cancer cells. Moreover, manoalide induces apoptosis, which are suppressed by NAC, MT, and ZVAD, suggesting that cellular and mitochondrial radical species can trigger apoptosis. Apoptosis induces cellular and mitochondrial reactive species, which are validated by the presence of ZVAD. It shows the reciprocally activation between reactive species (cellular and mitochondrial) and apoptosis. Therefore, we propose a mechanism of multifaceted inductions and interactions for cellular and mitochondrial reactive species to apoptosis on manoalide-treated oral cancer cells (Figure 9).

## Data Availability

No data were used to support this study.
